# Effectiveness of an mHealth App That Uses Financial Incentives and Gamification to Promote Health Behavior Change in Adolescents and Caregivers: Protocol for a Clinic-Based Randomized Controlled Trial

**DOI:** 10.2196/63505

**Published:** 2024-09-10

**Authors:** Amy Braddock, Parijat Ghosh, Emma Montgomery, Crystal Lim, Jaya Ghosh, Nicole Henry, Mihail Popescu, Kimberly Kimchi, Congyu Guo, K Taylor Bosworth, Richelle J Koopman

**Affiliations:** 1 Family and Community Medicine University of Missouri, Columbia Columbia, MO United States; 2 Department of Health Psychology University of Missouri, Columbia Columbia, MO United States; 3 School of Biomedical Engineering, Science & Health Systems Drexel University Philadelphia, PA United States; 4 Department of Chemical and Biomedical Engineering University of Missouri, Columbia Columbia, MO United States; 5 School of Medicine University of Missouri, Columbia Columbia, MO United States; 6 Biomedical Informatics, Biostatistics and Medical Epidemiology University of Missouri, Columbia Columbia, MO United States; 7 Department of Electrical Engineering and Computer Science University of Missouri, Columbia Columbia, MO United States

**Keywords:** mHealth, adolescents, apps, caregivers, obesity, healthy lifestyle, CommitFit, mobile health

## Abstract

**Background:**

Adolescent and adult obesity continues to be a public health epidemic in the United States. Despite the popularity of mHealth apps with gamification among adolescents, there are insufficient studies to evaluate the efficacy of gamified mHealth apps and financial incentives to motivate sustained health behavior change in adolescents or their adult caregivers.

**Objective:**

This study aims to evaluate the effectiveness of gamification techniques and financial incentives used in the novel “CommitFit” mHealth app to motivate health behavior change and improve various mental and physical health metrics in adolescents and their caregivers.

**Methods:**

This study is a 3-month randomized controlled trial (RCT) with 30 adolescents (aged 13-15 years) and their adult caregivers (N=60). It evaluates “CommitFit,” which uses gamification including points and leaderboards to motivate logging and achievement of self-selected health behavior goals (eg, more water, sleep, physical activity, fruits, or vegetables or fewer sugary beverages). The RCT had three arms, each with 10 dyads: (1) CommitFit-only users; (2) CommitFit$, where adolescents were paid US $0.05 for each point they earned; and (3) waitlist control. Intervention dyads used the app for 3 months and had the option to use it for the fourth month without prompts or extra financial incentives. User analytic software was used to evaluate the frequency of user logs and goal achievement. Monthly surveys evaluated self-reported change in the 5 CommitFit health behaviors. Changes in BMI and blood pressure were evaluated for all participants at 3 clinical visits. Mental health, gamification, and behavior economics surveys were completed during the clinical visits.

**Results:**

Recruitment began in August 2023 and was completed in 10 weeks. The research team successfully recruited and enrolled 30 dyads. Researchers emailed and called 89 caregivers on a physician-approved adolescent patient list, a 33% recruitment rate. Data collection and analysis will be conducted in the spring and summer of 2024. The results of this study are anticipated to be published between late 2024 and early 2025.

**Conclusions:**

This RCT will expand knowledge of the effectiveness of gamification techniques, financial incentives, and mHealth apps to motivate sustained health behavior change among adolescents and caregivers. These results may offer new opportunities to caregivers, health insurers, health care systems, and clinicians to motivate health behavior change in adolescents and caregivers, with the ultimate goal of preventing or reducing obesity and obesity-related diseases. Additional gamification, mental health surveys, and app user analytics included in the study may provide further insight into the characteristics of adolescents or caregivers who would benefit the most from using a gamified mHealth app like CommitFit.

**International Registered Report Identifier (IRRID):**

DERR1-10.2196/63505

## Introduction

### Background and Rationale

Adolescent obesity continues to be a significant public health problem in the United States. In 2020, 20% of children (aged 12-19 years) had obesity [1]. Adolescents with obesity are more likely to have obesity as adults [2], consequently facing an increased risk of developing obesity-related diseases such as heart disease, type 2 diabetes mellitus, hypertension, and some types of cancer [3].

Children with obesity are more likely to have parents or caregivers who also have obesity [4]. However, current obesity research generally focuses on the adolescent or caregiver with obesity independently and fails to design interventions that engage both the adolescent and caregiver. Family-based treatment is more effective for weight loss and other positive health outcomes [5]. An approach that includes both the adolescent and caregiver is more likely to result in weight loss for the adult caregiver [6]. Additionally, evidence supports bidirectional improvement; children lose more weight when their parents lose weight through family-based treatment [7]. Yet, there is a lack of studies that evaluate the inclusion of parents or caregivers in adolescent interventions [8].

mHealth apps offer a promising opportunity to address adolescent and adult obesity; however, there are significant deficits in the literature about how to best use this technology. An estimated 50% of the 3.4 billion global smartphone users have downloaded at least 1 mHealth app [9]. mHealth apps are a preferred intervention delivery method for adolescents because of their use of gamification or “game design elements in nongame contexts” [10], such as points, leaderboards, badges, avatars, and challenges to motivate health behavior change [9]. mHealth apps show promise for short-term reductions in adolescent weight [11] but have not been evaluated long-term or with concurrent use by adult caregivers [12,13]. Additionally, although 64% of American adolescents report having ever used an mHealth app, few report consistently using them [14], due to their lack of financial and gamification incentives to maintain their motivation and attention [15,16]. We developed the “CommitFit” mHealth app and conducted the current randomized controlled trial (RCT) to address these gaps.

The CommitFit mHealth app uses gamification techniques and financial incentives to motivate adolescents to set and achieve their health behavior goals. While the CommitFit mHealth app may improve adolescent and caregiver weights, it was intentionally designed not to be a weight loss app and does not ask users to log or track their weight. Rather it focuses on improving health behaviors for adolescents and adult caregivers. CommitFit was developed with significant adolescent, caregiver, and health care provider input received from focus groups, interviews, and a feasibility study in which the adolescent used CommitFit for 2 weeks and then provided qualitative and quantitative feedback regarding the app [17,18], all of which were integrated based on the user-centered design process [19].

This project will evaluate the use of the CommitFit app through a clinic-based RCT. Our primary objective is to evaluate the effectiveness of gamification techniques in CommitFit and financial incentives to motivate health behavior change in adolescents and their caregivers.

### Conceptual Framework

Behavioral economics (BE) seeks to understand why humans sometimes do not act in a rational fashion that is in their long-term self-interest [20]. Similar to intervention studies based on self-efficacy theory [21], previous BE-based intervention studies have been encouraging successful increases in physical activity and improved child nutrition [22]. Based on social cognitive theory [23], social ranking is a BE concept that uses peer-normative feedback to influence individual health behaviors [24]. CommitFit uses social ranking by allowing users to earn points through daily health behavior logging and self-selected goal achievement and then ranking users on a leaderboard based on these points.

Financial incentives or paying participants who achieve a predetermined goal in an effort to make the task more attainable [25] is another BE approach that has successfully increased physical activity [26] and resulted in short-term weight loss in adults [24]. mHealth apps offer a convenient and effective modality to evaluate BE concepts [27]. What remains to be ascertained is whether these concepts can be operationalized in an intervention aimed at modifying health behaviors, and whether adolescents and caregivers will use the mHealth app for a duration sufficient to enact meaningful health behavior changes.

### Study Aims

The aims and hypothesis are as follows.

- Specific aim 1: Evaluate the effectiveness of behavior change in adolescents using CommitFit for 3 months (primary outcome) compared to the waitlist usual care, and at the 4-month follow-up.
- Hypothesis 1: Compared to the waitlisted usual care group of adolescents, CommitFit users will have a greater improvement in their health behaviors at 3 months, and at the 4-month follow-up.
- Specific aim 2: Evaluate the effectiveness of behavior change in adult caregivers using CommitFit for 3 months (secondary outcome) compared to the waitlist usual care group, and at the 4-month follow-up.
- Hypothesis 2: Compared to the waitlisted usual care group of adult caregivers, CommitFit users will have a greater improvement in their health behaviors at 3 months, and at the 4-month follow-up.
- Specific aim 3: Evaluate the effectiveness of financial incentives (cash per point) to increase the frequency that adolescents log in and achieve their health behavior goals.
- Hypothesis 3: The CommitFit user group with the additional financial incentives (CommitFit$) will have a higher frequency of logging and achieving their health behavior goals compared to the group without incentives (CommitFit).

## Methods

### Study Design

**Figure 1:**
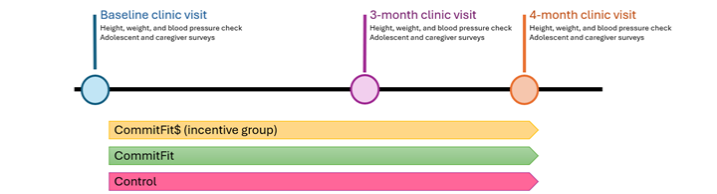
CommitFit randomized controlled trial.

We planned to conduct the 3-arm RCT with a total of 30 adolescents and 30 adult caregivers (30 dyads) of all weights at baseline. Participants were randomized into 3 arms, each with 10 dyads: CommitFit, CommitFit$ with an extra financial incentive, and waitlist control. The CommitFit intervention groups were encouraged to use the app consistently for 3 months. Adolescents in the CommitFit$ group were paid US $0.05 for each point they earned in the app, which the CommitFit group did not receive. After 3 months, both CommitFit groups had the option to continue to use the app if they chose but without extra financial incentives or encouragement from the research team. All participants returned for a 4-month visit, after which, control participants were given the option to download and use CommitFit (waitlist control; Figure 1).

### Study Setting

The study was conducted at the University of Missouri outpatient primary care clinic during after-school and work hours (from 4 PM to 7 PM). One of the principal investigators (AB) practices family medicine at this clinic and was able to obtain the support of clinic staff, nurses, leadership, and clinicians to conduct the study at this location. The initial enrollment visit (baseline) and 3- and 4-month follow-up visits all took place in private clinic exam rooms at this clinic with stadiometers, weight scales, and sphygmomanometer (blood pressure machine).

The CommitFit app, secured server, and user data analytic software were fully programmed and tested before and during the study with no major malfunctions. The principal investigators met with IT security and compliance officers to develop a plan regarding Health Insurance Portability and Accountability Act (HIPAA) compliance and IT security.

### Participants

The inclusion criteria for both caregivers and adolescents are as follows.

- Aged 13-15 years (adolescent) or over 18 years (adult caregiver)
- Speak English fluently and able to read at the sixth grade level or higher
- Self-reported proficiency with iOS Apple devices (eg, iPhone or iPad)
- Access to email for survey data collection (caregiver)

The exclusion criteria are as follows.

- Previous or current diagnosed eating disorders (adolescents or adult caregivers)
- Participants with self-reported severe or uncontrolled anxiety or depression (adolescent only; Participants with mild or controlled anxiety or depression were not excluded)

### Recruitment

**Figure 2:**
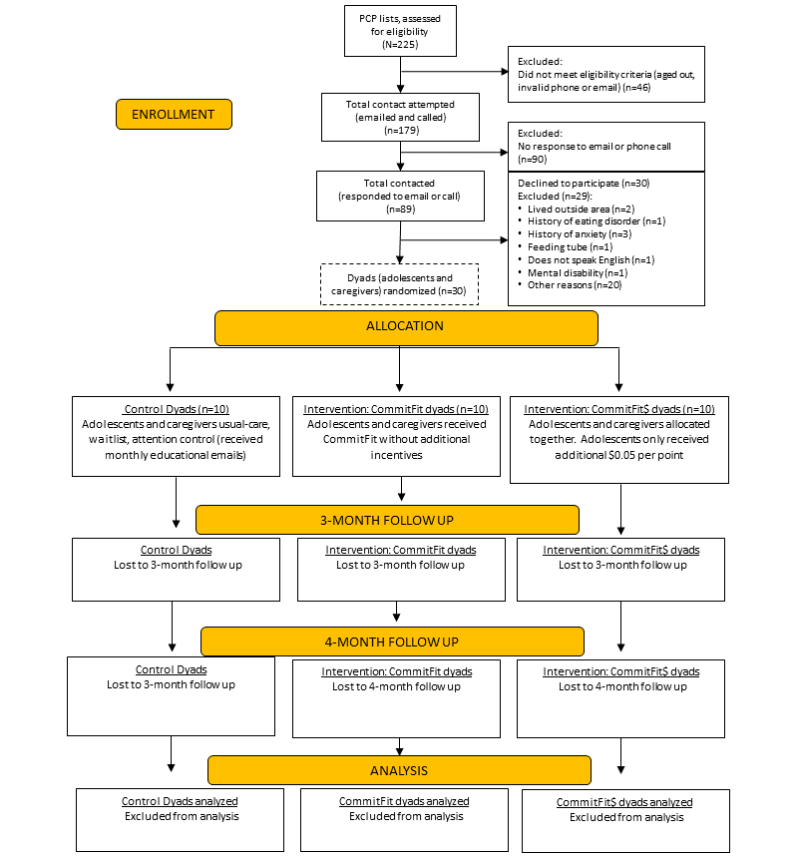
Research flowchart. PCP: primary care physician.

After obtaining a HIPAA waiver, researchers generated a patient list of adolescents aged 12-15 years followed by the University of Missouri family medicine and pediatric primary care physicians (PCPs) at the clinic. PCPs screened their list and removed any adolescents they did not believe would be appropriate for the study. All further recruitment was done by research staff to reduce the burden on clinicians and clinic staff. Adolescents who met age inclusion criteria and approval from PCPs and had their caregiver’s contact information in their electronic health record were emailed and then called (up to twice) by research staff to inform them of the study. An additional screening was conducted over the phone. Interested dyads who met screening criteria after the phone screening were scheduled for a baseline intake visit in the clinic where they completed informed consent and assent and enrolled in the study (Figure 2).

### Allocation

After being consented, adolescents were randomized into 1 of 3 arms (CommitFit, CommitFit$, or waitlist control groups) by selecting an opaque envelope with the assignment group printed on a piece of paper. Caregivers were assigned to the same arm as their adolescent, although caregivers did not receive the extra financial incentives (US $0.05/point) in the CommitFit$ group.

### Study Procedure

At each clinic visit, all adolescents and caregivers had their height, weight, and blood pressure (BP) recorded by the study team in private exam rooms or conference rooms. Nurses and health care providers provided the research staff with training on how to measure accurate blood pressures and weights on the clinic’s equipment, which included Seca stadiometers and scales (model 2841300109) and Welch-Allyn sphygmomanometers. The study team used the American Heart Association protocol [28] for checking and rechecking BP when indicated. BPs were checked with participants’ feet flat on the floor, after they had been sitting for several minutes, using an appropriately sized upper arm cuff with the BP cuff at heart level. Adolescent or adult participants with values consistent with stage 1 hypertension (≥130/≥80 mm Hg) [28] were notified by one of the principal investigators (AB), who is also a family medicine physician, and encouraged to follow up with their PCP. The PCPs were also contacted by the principal investigator (AB) to notify them of the elevated BPs.

In addition, participants completed electronic (via REDCap, Vanderbilt University) or paper surveys (Table 1) at each clinic visit. The caregivers and PCPs of adolescents who reported moderate or severe anxiety (score of greater than 10) on the 7-item General Anxiety Disorder [29] scale were notified by the principal investigator (AB) to follow up.

**Table 1:** Outcome measures.

Domain and measurement		Items, n	Respondent	Baseline	1 month	2 months	3 months	4 months
**Anthropometrics**								
	BMI%/BMI (height, weight)	2	Caregiver and adolescent	✓			✓	✓
	Blood pressure	1	Caregiver and adolescent	✓			✓	✓
**Health behaviors**								
	5 CommitFit behaviors	5	Caregiver and adolescent	✓	✓	✓	✓	✓
**Modifiers**								
	Demographics		Caregiver and adolescent	✓				
	Behavioral economics survey	5	Caregiver and adolescent	✓				
	Gamification survey	7	Caregiver and adolescent	✓				
**Health psychology surveys**								
	GAD-7^a^	7	Adolescent	✓			✓	✓
	PedQL^b^	11	Adolescent	✓			✓	✓
	Reasons for health	9	Adolescent	✓			✓	✓
**CommitFit surveys**								
	CommitFit use	8	Caregiver and adolescent, intervention groups only				✓	
	Commercialization	6	Caregiver and adolescent, intervention groups only				✓	

^a^GAD-7: 7-item General Anxiety Disorder.

^b^PedQL: Pediatric Quality of Life Inventory.

### CommitFit mHealth App

**Figure 3:**
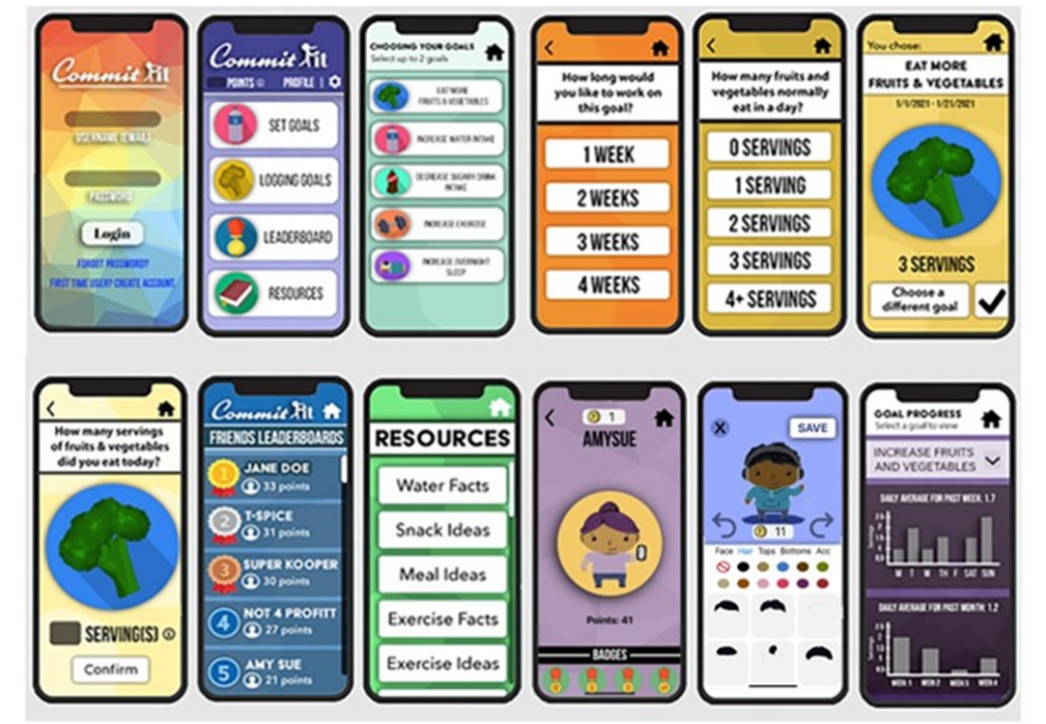
Selected screenshots of CommitFit app.

The CommitFit app (Figure 3) uses gamification techniques to motivate adolescents and caregivers to set and achieve health behavior goals. An in-app tutorial teaches users how to choose a health behavior goal (eg, eat more fruits or vegetables, increase water intake, decrease sugary beverage intake, increase physical activity, or increase overnight sleep), set a time period for that goal, and set a target amount (level) within that goal behavior. The app asks for the user’s current level of the specific targeted behavior and makes a modest recommendation to set the behavior goal 2 levels above their current level. For example, an adolescent who reported consuming no fruits or vegetables currently per day would be recommended to consume 2 servings of fruits or vegetables per day. Users are not required to follow this recommendation; however, they must set a behavior goal level equal to or higher than their current level but below a maximum threshold based on current clinical and evidence-based guidelines [30], aiming to prevent excessive behaviors. Users can choose up to 2 health behaviors to work on at once and can work on the same goal multiple times during the study period if they choose. Once a health behavior has been optimized (the user has chosen and achieved the recommended level for that health behavior for 2 goal periods), the user will be required to choose a different goal. The user can choose to work on a goal for 1, 2, 3, or 4 weeks. They will receive a reminder, which can be turned off or modified, in the morning and afternoon to log their health behavior. Users receive 1 point for each daily goal they log, an extra 1 point if they achieve the user-selected goal level, 6 points when they complete their goal period, and 10 points if they achieve, on average, the goal level at the end of the goal period. Users are then ranked by total points and points per week (to allow new users to be competitive). Families have the option to form teams and can also see their team points (averaged by 2 team members). Users earn CommitFit coins through the same methods which can be used to unlock gear and clothing (headphones and jewelry) for their customizable avatars (Figure 3). A variety of skin colors, hair textures, and style options for the avatars were intentionally included in the CommitFit app. In addition, CommitFit provides educational resources to support users in achieving their health behavior goals, and a weekly and monthly progress graph of their logged behaviors.

### CommitFit Group

Ten of the adolescents and their caregivers were randomly selected to use the CommitFit app, as described above, with full use of gamification features such as points and leaderboard, but without extra monetary incentives. All groups were compensated US $50 e–gift cards, which were emailed to each adolescent and caregiver after each clinic visit. Participants in the CommitFit group were assisted in downloading the CommitFit app through the TestFlight service (Apple, Inc), guided through logging in, and were provided training in how to use the app, including picking their first goal, setting a behavior level and time period (1-2 weeks for the first time period), logging their behavior daily, and setting notifications. Participants were educated on the leaderboard, avatar, progress report, gear store, and resources. A handout was also provided to each family with instructions on the functions of the CommitFit app.

Participants in the CommitFit group were asked to use the app consistently for 3 months, and they were allowed to continue to use CommitFit before and after their 3-month visit if they were interested in doing so. However, app use after the 3-month visit was not emphasized. There were no penalties if they did not use the app for the entire duration or used it infrequently.

If participants did not have access to an iOS device, the study team provided them with 1 loaner iPad and charger per family at the first clinic visit. Participants were asked to return the iPad and charger at their 4-month clinic visit to receive their last e–gift cards.

### CommitFit$ Group

Ten randomly selected adolescents and their caregivers used the CommitFit app just as the CommitFit group. In addition to the participant compensation of e–gift cards for completing clinic visits, adolescents in the CommitFit$ group received an extra US $0.05 for every point they earned using the app during the 3-month period. The maximum amount an adolescent can earn in a 3-month period is US $52.50 (equivalent to 1050 points). The purpose of this extra financial incentive is to evaluate if an external financial reward will provide enough motivation to encourage adolescents to log their daily health behaviors and achieve their health behavior goals. No extra financial incentives were given at the 4-month visit to determine how often the CommitFit$ adolescents would log and achieve their goals when the financial incentives were no longer provided.

The research team decided to provide additional financial incentives exclusively to the adolescents in the CommitFit$ group, rather than also to the caregivers. This decision was based on the likelihood that adolescents would be more motivated by such incentives. Additionally, there is a potential business model where caregivers could pay to incentivize positive health behaviors for their children. Further, we were interested to see how giving financial incentives solely to adolescents, not caregivers, would influence caregiver logging and goal achievement.

### Control or Waitlist Usual Care Group

The control dyads (n=10, 30%) received monthly emailed handouts with healthy lifestyle recommendations that are available and provided as electronic or paper handouts in the primary care clinics as an attention control. Topics for these handouts included healthy fruits and vegetables, tips on physical activity for teens, tips on sleeping habits, and tips on healthy eating. All groups were instructed to continue their routine medical care. After the 4-month clinic visit, the waitlist control group was provided the link and assistance to download and use the CommitFit app, if desired.

### Clinic Visits

Thirty dyads from all 3 arms (N=60) were scheduled to be seen for three research clinic visits: (1) baseline visit for screening, consent or assent, and randomization (caregivers completed a demographics survey for themselves and their adolescents at the baseline visit); (2) 3-month visit after CommitFit and CommitFit$ groups had used the app for 3 months; and (3) 4-month follow-up clinic visit.

### Outcomes

#### Primary Outcome

Our primary outcome was to evaluate changes in the 5 health behaviors that were promoted in the CommitFit app (fruits or vegetables, water, physical activity, decreased sugary beverages, and sleep) in adolescents and caregivers. Between the initial baseline and 3-month clinic visit (at 1 month and 2 months), caregivers were emailed surveys for themselves and the adolescent to fill out which consisted of the 5 CommitFit target health behaviors. Adolescents and caregivers completed the CommitFit behavior surveys in person during the clinic visits at baseline, 3 months, and 4 months. This allowed for monthly assessments of CommitFit health behavior changes in adolescents and caregivers.

#### Secondary Outcome

Our secondary outcome was to determine if the additional financial incentives received by CommitFit$ groups increased user daily logging and achievement of their health behavior goal. These data were collected internally through user analytic software programmed by the research team. The research team received monthly reports of each activity conducted by each user including when a goal was set, what the goal was, each time the user logged a health behavior, and the level of that logged behavior. The research team was able to use this user data to determine the percentage of days the user logged at least 1 behavior (% logged) and the percentage of days the user achieved their self-selected health behavior goals (% goal achieved).

#### Additional Anthropometric Outcomes

Height and weight for the adolescent and caregiver were measured by the study team at their baseline and the 3- and 4-month clinic visits, and these were used to calculate BMI% for the adolescent and BMI for the caregiver. These metrics were used over other weight metrics because they have been shown to be highly correlated with adiposity change in children over time [31,32]. BP was also measured at each clinic visit for adolescents and caregivers.

#### Additional Surveys

At baseline, all participants completed behavior economics and gamification surveys created by the research team to evaluate the participants’ self-awareness of potential motivating factors. Other mental health surveys included the 7-item General Anxiety Disorder, which evaluates symptoms of anxiety; the Pediatric Quality of Life Inventory, which assesses health-related quality of life [33]; and a Reasons for Health survey, which the research team created to evaluate adolescent’s motivation to be healthy. These surveys were completed at baseline, 3 months, and 4 months by adolescent participants.

### Ethical Considerations

#### Institutional Review Board Approval

This study was approved by the University of Missouri Health Science Institutional Review Board (2092610). This RCT has not been registered in a trial registry because it was not required by the funder based on the small sample size and exploratory nature of the study.

#### Minimizing the Risk of Harm

The harm associated with weight-based discomfort or eating disorders was minimized in the CommitFit app by focusing on behavior change strategies rather than weight or calorie restriction. CommitFit was programmed to set limits on health behavior goals to prevent extreme or unhealthy goal-setting and behaviors. CommitFit does not collect or display any personal or sensitive information from users. The risk of harm associated with undiagnosed or untreated anxiety or hypertension was mitigated by contacting the caregiver and the user’s PCP.

The potential risk of harm from an accidental breach of personal health information and data was minimized by removing identifying information before analyzing, sharing, or publishing any results. We maintained the participant’s data and participation status confidentially on a password-protected laptop and server. All potential risks were disclosed on the consent forms.

#### Consent

The study team obtained consent by having the caregiver review and sign consent forms for themselves and their child. Additionally, the adolescents reviewed and signed the child assent form for themselves. Initially, we attempted to obtain signed consent before the baseline clinic visit, via Docusign (Docusign, Inc). However, this was logistically challenging, and after the first few dyad clinic visits, all consents were obtained in-person at the in-person baseline clinic visit. This allowed the research staff to explain the study and answer questions in person. Participants were provided electronic copies and offered paper copies of all consent forms.

#### Compensation

All study participants (both adolescents and caregivers) each received a total of US $150 e–gift cards for full participation in the study. The planned study protocol was to provide two US $50 e–gift cards (one for the caregiver and one for the adolescent) to be sent to the caregiver’s email after each of the 3 clinic visits. However, due to an administrative error, the first 2 rounds of e–gift cards (4 e–gift cards) were sent out after the first clinic visit. The study protocol was thus changed to require participants to attend the 3-month and 4-month clinic visits to receive their last two US $50 e–gift cards. Despite this protocol deviation, attendance at the 3- and 4-month clinic visits was still high.

### Data Management

All surveys completed during clinic visits were either conducted on laptop computers directly into RedCap or, if this was not available, through paper surveys and then entered by research staff into RedCap. Monthly CommitFit health behavior surveys were generated and emailed through RedCap for months 1 and 2. Paper surveys and consent forms were stored in locked research or clinic offices and destroyed after being digitally stored. Results were stored and shared with the research team using password-protected Microsoft Teams folders on network-secured servers. All databases are HIPAA compliant. The principal investigator will maintain electronic copies of data that have been deidentified on password-protected, network-secured laptops for 10 years or until all related publications and submissions have been completed.

### Data Analysis Plan

Descriptive statistics were used to examine baseline demographics and will be used to examine study outcomes over time. Chi-square analysis will be used to compare group differences for categorical outcomes and 2-tailed *t* test or ANOVA for continuous outcomes. Cronbach α will be used when evaluating the internal reliability of study-developed scales. All analyses will be conducted using SAS version 9.4 (SAS Institute).

## Results

The research team successfully recruited and enrolled 30 dyads (60 total adolescents and caregivers) in 10 weeks (from August 2023 to October 2023) by emailing and calling 89 caregivers of PCP-approved adolescent patient lists, achieving a 33% recruitment rate. Data collection, including CommitFit user analytics, and statistical analysis of primary and secondary study outcomes are being conducted in the spring and summer of 2024. The results of this study are anticipated to be published in late 2024 and early 2025.

## Discussion

### Expected Outcomes and Potential Impact

The purpose of this study was to evaluate the effectiveness of the CommitFit mHealth app, which uses gamification and financial incentives to motivate sustained health behavior change in adolescents and adult caregivers. The results from this study will inform future mHealth lifestyle and adolescent and adult obesity interventions. It will help researchers and app developers design programs that appeal to users and sustain their attention and motivation long enough to result in sustained health behavior changes and improved health outcomes. This RCT was also designed to evaluate the influence of including an adult caregiver with the adolescent mHealth intervention.

### Dissemination Plan

The results of the study will be disseminated through publications, presentations, and meetings with stakeholders. We will use contacts through our advisory boards and funders to identify key stakeholders to disseminate the results of this and future studies to facilitate the further development and dissemination of CommitFit. With the assistance of these stakeholders, we will identify additional funding and resources to support implementation and dissemination.

### Limitations

This study has some limitations. Anthropometrics (BMI%, BP) were collected in the study. However, because most of the adolescents included in the study had a healthy weight at baseline (overweight or obesity was not an inclusion criterion in this study) and because of the small sample size, a significant improvement in these measurements is not anticipated in adolescents or caregivers. Our next fully scaled study will only include adolescents and caregivers with overweight or obesity and will be conducted for 6 months to evaluate the effectiveness of CommitFit to improve BMI over an extended period of time.

Two server errors occurred during the study, which caused the CommitFit app to be unusable. The first occurred around 1 month and another close to 3 months after the study had begun. The first server error lasted a few days and the second lasted a day. Within 8 hours of the server error occurring, all participants in the intervention groups were emailed regarding the malfunction, steps to mitigate it, and a request to physically log their health behaviors. After server errors were fixed and the app was running again, the study team sent a follow-up email to all participants in the intervention groups letting them know the app was working again and a request to email the study team their health behaviors so their total points could be recorded, and the appropriate financial incentives could be provided to the CommitFit$ adolescents at the end of the study. Because the server errors lasted a short time relative to the duration of the study (4/120 days) and because we manually recorded the logged health behaviors that participants emailed to the study team, we do not feel these server errors significantly affected data collection or the intervention; however, we will evaluate this when conducting primary analyses. The rapid and thorough response to the server errors seemed to be appreciated by the participants.

### Strength

We attribute the high recruitment rate (N=60) in a short period of time (11 weeks) to a strong recruitment strategy. Two of the principal investigators are family medicine physicians with good relationships with the PCPs, nurses, and clinical staff where the study visits occurred. This helped to expedite the PCP approval of the prescreened adolescent recruitment list. Researchers also leveraged the relationship between the PCP and participants when recruiting them for the study. For this research strategy to be effective, trust must be maintained between the participant, the research team, and the PCP. Researchers should consider collaborating with clinicians for their clinical research studies.

Because it takes time for recruitment lists to be generated, for institutional review board amendments to be approved, and for study startups to occur, and because adolescents on the original list may age out of the inclusion age range, the research team found it useful to include adolescents a year younger than the inclusion criteria in the initial list (aged 12-15 years, rather than 13-15 years) and to check the adolescent’s current age before contacting their caregiver for recruitment. Once the recruitment list was approved by the PCP, the research team found that the most effective recruitment method was to email caregivers, wait 2 days, then call them, up to twice. Including both the adolescent and caregiver in the study and providing both with compensation individually also seemed to increase recruitment.

This RCT will expand knowledge regarding the use of gamification and BE techniques including financial incentives to motivate health behavior change among adolescents and their caregivers. These results may offer new opportunities to parents or caregivers, health insurers, health care systems, and clinicians to motivate health behavior change in adolescents and caregivers, with the ultimate goal of preventing or reducing obesity and obesity-related diseases and morbidities. Additional gamification, BE, mental health surveys, and app use analytics included in the study may provide additional insights into the characteristics of adolescents or caregivers who would most benefit from using a gamified mHealth app like CommitFit.

## Appendix

Multimedia Appendix 1 Peer review report by the Oversight Committee - University of Missouri (MU) Coulter Biomedical Accelerator (USA).

## Abbreviations

BE: behavioral economics
BP: blood pressure
HIPAA: Health Insurance Portability and Accountability Act
PCP: primary care physician
RCT: randomized controlled trial

## Data Availability

The datasets generated or analyzed during this study are available from the corresponding author upon reasonable request.
