# Supplementary material for: Effectiveness of an mHealth App That Uses Financial Incentives and Gamification to Promote Health Behavior Change in Adolescents and Caregivers: Protocol for a Clinic-Based Randomized Controlled Trial
Source: JMIR Res Protoc. 2024 Sep 10;13:e63505. doi: 10.2196/63505 (PMC11422729; doi:10.2196/63505)
Supplement: Multimedia Appendix 1 [file resprot_v13i1e63505_app1.pdf]

Coulter Biomedical Accelerator
University of Missouri

July 25, 2022

Dear Team CommitFit,

Congratulations! I am pleased to inform you that the MU Coulter Program has voted to fully fund your project in tranche-based milestones. We will start by funding your Milestone 1 ($24,050) – as communicated during your LOI submission, funding for Milestone 2 and subsequent milestone(s) will be contingent upon successful + timely completion of previous milestone(s), and submission of progress report(s) to MU Coulter for review. Below are additional requests/overall feedback:

- Documents Required: IRB approval/amendment.
- Feedback:
    - Follow-on funding: Explore NIH clinical trial opportunities. MO Medicaid should be one of several strategies to pursue.
    - Commercialization support required.
    - Intellectual Property: As you work on your Coulter milestone(s), please meet with MU TAO on a regular basis, and keep me posted regarding your IP status. At this stage, it is understood that software copyright is possible.
    - Consider partnering with school systems.

I would like to set up a time to discuss the specific conditions, share the Committee's detailed feedback with you, and also go over the Terms and Conditions of Coulter funding. Please let me know some dates/times that work for the team during the weeks of 8/8 and 8/15. Again, congratulations! 😊

Thank you.
Sincerely,

Jaya Ghosh, PhD *(she/her)*
Program Director | MU Coulter Biomedical Accelerator
Lead Program Manager | Midwest Biomedical Accelerator Consortium (MBArC), NIH Research Evaluation and Commercialization Hub (REACH)
Associate Director | Midwest BioAccelerator, MU BARDA DRIVe Accelerator
Adjunct Professor | Bioengineering, College of Engineering
University of Missouri
C3216 Lafferre Hall
Columbia, MO 65211
573.882.0522 (office)
Website: Coulter | MBArC

Coulter Biomedical Accelerator

University of Missouri

Below is the University of Missouri Coulter Biomedical Accelerator Oversight Committee's feedback on your pitch presentation.

Summary: The clinical need being addressed in this application is significant and the proposed pilot study, if successfully completed, could generate data and insights that would be critical in designing future clinical trials. While the novelty of the App is moderate, the individualized approach to serving the target market is a strength. The team is highly motivated but requires extensive support and mentorship in developing their Intellectual Property position and get-to-market strategy.

Reviewer 1	• Big unmet need. Need to look at market and potential buyer. Need more input for potential business.<br>• Worth developing app.<br>• Is Weight Watchers for kids possible?
Reviewer 2	• Definite need.
Reviewer 3	• Small market size. Compliance and ongoing use likely a challenge. Barriers to entry low.
Reviewer 4	• The unmet need is real, and an app designed for adolescents makes sense and is more likely to be effective.
Reviewer 5	• Interesting app but they would need lots of marketing help.
Reviewer 6	• Great team. I have concerns about this app becoming a commercial product.<br>• I like the idea of supporting this project in some way, in hope that an app could be developed and tested in a clinical trial.
Reviewer 7	• Good opportunity to develop a prototype that might be used in broader federal funding... beyond MO Medicaid.<br>• Apply for external funding, hopefully NIH.
Reviewer 8	• My main concern is that this project as defined will not produce any future income to MU. The copyright in and of itself has no commercial value. My other concern is the lack of experience within the team to develop a consumer application. I would rather see this effort continue via grant funding as a research project.
Reviewer 9	• Business Opportunity: Good idea but the chance it will make money is very limited – unless they are acquired by a weight loss company (like Weight Watchers). The enhanced program cost/week is very unrealistic. I worked in diabetes disease management and faced these challenges in the past. Most potential diabetic users (who can lose a foot) wouldn't pay and payers would not invest in outcomes that may come in several years. The revenue projections will be a challenge.

Reviewer 10	• A very enthusiastic team. Reimbursement is the main unknown. They didn't come up with really a fresh idea that would give them an advantage in attracting users, but it's hard to know what may click with adolescents.
